# Knowledge about Health Effects of Cigarette Smoking and Quitting among Italian University Students: The Importance of Teaching Nicotine Dependence and Treatment in the Medical Curriculum

**DOI:** 10.1155/2014/321657

**Published:** 2014-04-06

**Authors:** Maria Caterina Grassi, Massimo Baraldo, Christian Chiamulera, Franco Culasso, Tobias Raupach, Amy K. Ferketich, Carlo Patrono, Paolo Nencini

**Affiliations:** ^1^Department of Physiology and Pharmacology “V. Erspamer”, School of Medicine, Sapienza University of Rome, Piazzale Aldo Moro 5, 00185 Rome, Italy; ^2^Department of Experimental and Clinical Medicine, School of Medicine, University of Udine, Piazzale S. Maria della Misericordia, 33100 Udine, Italy; ^3^Department of Public Health and Community Medicine, Section of Pharmacology, University of Verona, Policlinico G. B. Rossi, Piazzale Scuro 10, 36134 Verona, Italy; ^4^Department of Public Health and Infectious Diseases, School of Medicine, Sapienza University of Rome, Piazzale Aldo Moro 5, 00185 Rome, Italy; ^5^Department of Cardiology and Pneumology, University Hospital Göttingen, Robert-Koch-Strasse 40, 37075 Göttingen, Germany; ^6^Department of Epidemiology and Public Health, Health Behaviour Research Centre, University College London, 1-19 Torrington Place, London WC1E7HB, UK; ^7^Division of Epidemiology, The Ohio State University College of Public Health, 1841 Neil Ave, 310 Cunz Hall, Columbus, OH 43210, USA; ^8^Department of Pharmacology, School of Medicine, Catholic University of Rome, Largo F. Vito 1, 00168 Rome, Italy

## Abstract

Aims of the study were to compare medical students (MS) to non-MS with respect to their knowledge of smoking and to investigate the effect of a short educational intervention on MS knowledge. MS (*n* = 962) and students of architecture and law (*n* = 229) were asked to complete a 60-item questionnaire addressing knowledge of smoking epidemiology and health effects (“Score 1”), and effectiveness of cessation treatments (“Score 2”). Upon completion of questionnaire, fourth year MS received a lecture on tobacco dependence. These students were asked to complete the same questionnaire one and two years later. Mean values for Score 1 were 48.9 ± 11.5% in MS and 40.5 ± 11.4% in non-MS (*P* < 0.001; *d* = 0.69). Respective values for Score 2 were 48.1 ± 10.8% and 42.6 ± 10.6% (*P* < 0.001; *d* = 0.50). Fifth year students who had attended the lecture in year 4 scored higher than students who had not attended the lecture. Significant differences were noted one but not two years after the educational intervention. In conclusion, MS know slightly more about smoking-related diseases and methods to achieve cessation than nonmedical students; a short educational intervention was associated with better knowledge one year later, but the effect was moderate and short-lived.

## 1. Introduction

Tobacco smoking is the leading cause of preventable death in developed countries and is the most important risk factor for cancer worldwide, responsible for approximately 22% of all cancer deaths per year [1, 2]. According to the Osservatorio Fumo, Alcol e Droga, about 11 million adults in Italy are still current smokers, 20.7% of the entire adult population [3]. Smoking is the largest avoidable health risk in Europe, causing more problems than alcohol, drugs, high blood pressure, excess weight, or high cholesterol (http://ec.europa.eu/health/tobacco/policy/index_en.htm). Consequently, every year, 695,000 Europeans die prematurely of tobacco-related diseases and it is estimated that, within the EU, smoking causes annual costs of at least €100 billion (http://ec.europa.eu/health/tobacco/docs/eurobaro_attitudes_towards_tobacco_2012_en.pdf). Conversely, smoking cessation reduces health risks and improves quality of life. In particular, the cumulative risk of dying of cancer, cardiovascular and lung diseases can be drastically reduced if smokers quit, even at an advanced age [4–6]. There is no doubt that medical advice helps smokers quit [7], yet often this opportunity is missed [8–10]. The frequent observation of general practitioners (GPs) not adhering to guidelines for brief counseling might at least partially be due to inadequate training in undergraduate education. Indeed, substantial deficiencies in medical education on smoking-related issues have been described [11–15]. This is not surprising since little attention is being paid to nicotine dependence in medical school curricula; a worldwide survey recently revealed that only one in four medical schools taught a specific module on nicotine dependence [16].

Recent studies on medical education in various European countries have consistently shown that undergraduate training in this area is insufficient. This is surprising when considering that well-conceived educational interventions to improve knowledge, skills, and attitudes of medical students regarding the treatment of smokers are available [17, 18]. Arguably, one factor limiting the implementation of such programs is their high cost in terms of resources and teacher time. Therefore, there is a need for straightforward and relatively simple but yet effective tobacco curricula. For instance, even one single lecture on the topic might be enough to stir the interest of students eliciting self-directed learning activities with regard to tobacco toxicology and treatment options. More high-quality research in this area is clearly needed [19].

We recently reported that Italian students attending the fourth year of undergraduate medical education have limited knowledge about tobacco dependence, smoking-related pathologies, and the role of physicians in promoting smoking cessation [20]. While these findings in themselves are a cause for concern, their interpretation might be further enhanced by comparing them to survey results obtained from nonmedical students. Since medical education needs to prepare future physicians for their role as health advocates, one would expect medical students to know substantially more about smoking and cessation than students of nonmedical professions. However, to the best of our knowledge, nonmedical students have rarely been surveyed with regard to their knowledge about tobacco.

Based on these considerations, the aims of this study were to (i) verify the consistency of our previous findings [20], (ii) assess whether nonmedical students of the same age have different perceptions and knowledge about smoking compared to medical students, and (iii) monitor knowledge retention of tobacco dependence and medical students smoking status, one and two years following a short educational intervention.

## 2. Methods

### 2.1. Questionnaire

Students were asked to complete a 60-item questionnaire, previously validated [20], derived from studies on this topic [21, 22]. The questionnaire was composed of four main sections:

- demographics and personal smoking history: gender, age, age at initiation, cessation history, intention to quit, and nicotine dependence using the Fagerström Test for Nicotine Dependence (FTND) [23],
- knowledge of smoking-related epidemiologic facts: knowledge of smoking attributable mortality, tobacco toxins, health risks associated with smoking, and the benefits of smoking cessation,
- knowledge of clinical guidelines on tobacco dependence treatment, as well as competence in counseling a smoker seeking help to give up smoking,
- perception of the influence of smoking on life expectancy: students were asked whether they personally knew smokers and nonsmokers who had lived to the age of 90 years (total 2 questions). One further question was asked about knowledge of tobacco treatment centers in the city of their university and one final question was asked to students, “Would you like a smoke-free university?” Response options were “yes” or “no.”

To assess the knowledge of tobacco and cessation, two scores were computed. “Score 1” was based on responses to questions on the epidemiology of smoking and related risks, as well as on the benefits of quitting smoking; “Score 2” was based on responses to questions about nicotine dependence treatments and their effectiveness (see data analysis for the details of scores computing). For a detailed description of the questionnaire, see our previous paper [20]. The questionnaire is available upon request.

### 2.2. Study Participants

Students from four different medical schools and one school of architecture and law were invited to participate in the study. Two of the four medical schools (Sapienza University of Rome, Catholic University, Rome Campus) as well as the Schools of Architecture and Law (Sapienza University of Rome) were located in Rome whereas the other two medical schools were located in Northern Italy (University of Udine and University of Verona). All medical schools involved offered a standard core curriculum representative of that given by other medical schools in Italy, in which drug addiction is a topic of the fourth year. In this year preclinical courses are dealing with general and specific health risks including cigarette smoking. In order to address the three study aims, students were divided into eight different groups as described below.

*Study Question  1*. “Does the questionnaire produce consistent results in consecutive cohorts of medical students?” To answer this study question, fourth year medical students from two consecutive cohorts (2010: Group 1; 2011: Group 2) were invited to participate in the study. Students in both cohorts were enrolled in the course of pharmacology and toxicology and completed the questionnaire before attending a lecture on nicotine dependence.

*Study Question  2.* “Do nonmedical students of the same age have different perceptions and knowledge about smoking compared to medical students?” To answer this study question, two cohorts of fourth year students studying architecture (Group 5) and law (Group 6) were invited to complete the study questionnaire in 2012 and were compared with fourth year medical students (Group 1 and Group 2).

*Study Question  3.* “How much knowledge of nicotine dependence is retained by medical students one and two years following a short educational intervention?” To answer this study question, the 2010 student cohort was followed up for 2 years (2011: Group 3; 2012: Group 4). Each year, students completed the same questionnaire. While doing so, they were asked whether they had attended the lecture in 2010. Based on their replies, students were labelled as being in the “control” (neither questionnaire nor lecture: Groups 3A and 4A) or “intervention” (questionnaire + lecture: Groups 3B and 4B) cohorts.

**Figure 1:**
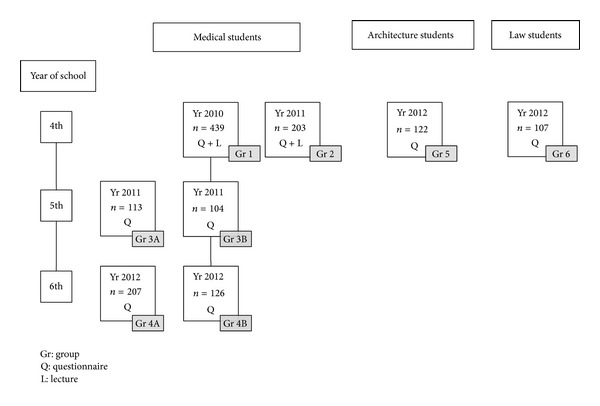
Chart of the different groups of 1191 students enrolled in the study according to year of school, university school, and intervention on nicotine dependence (questionnaire and lecture or questionnaire only).

For more information on the flow of participants through the study and sample sizes, please see Figure 1.

### 2.3. Data Collection

Between April 2010 and November 2012, students attending the academic courses were invited to complete the questionnaire; participation was voluntary and anonymous. After having explained the purpose of the study, a pharmacology lecturer distributed the questionnaire and students were allowed 30 minutes to complete it. Lecturers remained in the room but kept at a distance from participating students in order to ensure anonymity of the responses. In the case of students attending the course of pharmacology and toxicology, offered only to the fourth year in all participating medical schools, a teaching lecture based on specific protocol, dealing with epidemiology of smoking-related diseases, health risk of smoking, and nicotine dependence and its treatment, was delivered by the pharmacology teacher, after questionnaire completion.

### 2.4. Data Analysis

As previously described [20], the questionnaire contained 46 close-ended questions and 1 open-ended question, for a total of 60 items, since some questions consisted of more than 1 item. Ten questions (14 items) were used to calculate Score 1 whereby each answer was assigned a value between 0 and 2 (range 0–28). A value of 2 implied that the students answered correctly, a value of 1 implied that the answer was in the 10% range of a quantal response, and a value of 0 implied a totally incorrect answer. Questions that were not answered were counted as incorrect answers. The items for Score 1 included (i) smoking epidemiology; (ii) risks associated with smoking; and (iii) benefits of cessation. Using nine additional questions (14 items), another Score 2 was computed, assigning a value of 0 to 2 to each answer (same mechanism for assigning values for Score 1), to evaluate students based on their knowledge of (i) clinical guidelines on smoking cessation; (ii) effectiveness of smoking cessation methods.

Descriptive statistics were performed for each question. Since all data were collected anonymously, we were unable to match individual student data obtained in the 2010 cohort to subsequent surveys in this longitudinal cohort. Thus, all groups were treated as independent groups, and analysis of variance (ANOVA) was performed to assess significant differences between groups, followed by post hoc Bonferroni corrections for study questions 1 and 3. Student's *t*-test was used to compare mean values obtained from the two groups (study question 2). Cohen's *d* effect sizes were calculated.

For dichotomous variables, chi-square tests were performed. Differences were considered statistically significant at a *P* value <0.05. Statistical analyses were performed using SPSS Version 20.0 for Mac.

Approval of the study method was obtained from the Ethics Committee of the Hospital Policlinico Umberto I, at Sapienza University of Rome, as well as from the Dean of each of the other participating medical and nonmedical schools.

## 3. Results

The questionnaire was completed by 1191 students, 962 of whom were medical students (61% female, mean age 23.9 ± 2.8 years, range 20–55), 122 studied architecture (57% female, mean age 23.2 ± 3.4 years, range 20–41), and the remaining 107 studied law (72% female, mean age 21.7 ± 2.0 years, range 20–38). All the students present in the class agreed to complete the questionnaire. Questionnaire completion was satisfactory, as the response rate was of 100%, missing items were fewer than 10%, and the proportion of missing values did not differ significantly between groups.

### 3.1. Demographic Characteristics, Personal History of Tobacco Use, and Intention to Quit

As shown in Table 1, self-reported current smoking was significantly higher (*P* < 0.01) among architecture (26.2%) and law (26.2%) students compared to medical students (16.9%). Among the latter, the percentage of current smokers was significantly higher in males than females (22.0% versus 13.6%; *P* = 0.001). A similar gender difference was observed among architecture and law students, although statistical significance was not reached (architecture: 29.4% versus 25.4%; law: 33.3% versus 24.0%). Smoking students scored low on the FTND, and the majority (66.8%) smoked less than 10 cigarettes per day and wanted to stop smoking (57.9%). A particularly low smoking prevalence was noted in the 6th year medical students who had attended the lecture on nicotine dependence during their fourth year (2010 cohort; Group 4B). Their smoking prevalence of 10.3% was less than half of that found in sixth year students who had missed the lecture (Group 4A: 23.7%; *P* = 0.001). Only one-fifth of the smoking students (22.3% of medical and 18.3% of nonmedical students) reported having received advice to stop smoking by a GP during the past year.

### 3.2. Smoking and Life Expectancy, Wishing a Smoke-Free University, and Knowledge of Tobacco Treatment Centers

As expected from our previous work [20], we found that the percentage of medical students claiming they personally knew a smoker who had lived to the age of 90 years was significantly greater in smokers than in nonsmokers (55.8% versus 39.8%; *P* < 0.01), whereas the percentage of students answering that they personally knew a nonsmoker who had reached the age of 90 years was similar in smokers and nonsmokers (87.3% smokers versus 87.1% nonsmokers) with no statistically significant differences between the six groups considered.

The vast majority of nonsmoking medical students (91.4%) claimed they would like to study in a smoke-free university while this view was only supported by 48.2% of smokers (*P* < 0.001). The corresponding figures for architecture and law students were 78.9% versus 15.6% (*P* < 0.001) and 79.7% versus 35.7% (*P* < 0.001), respectively.

Finally, 40.4% of fifth year medical students in Group 3B, 16.7% of sixth year medical students in Group 4B, 11.3% of those in Groups 1, 2, 3A, and 4A, and only 2.6% of nonmedical studentswere aware of the existence of tobacco treatment centers in the city of their university.

### 3.3. Comparisons between Student Groups

#### 3.3.1. Study Question 1

**Figure 2:**
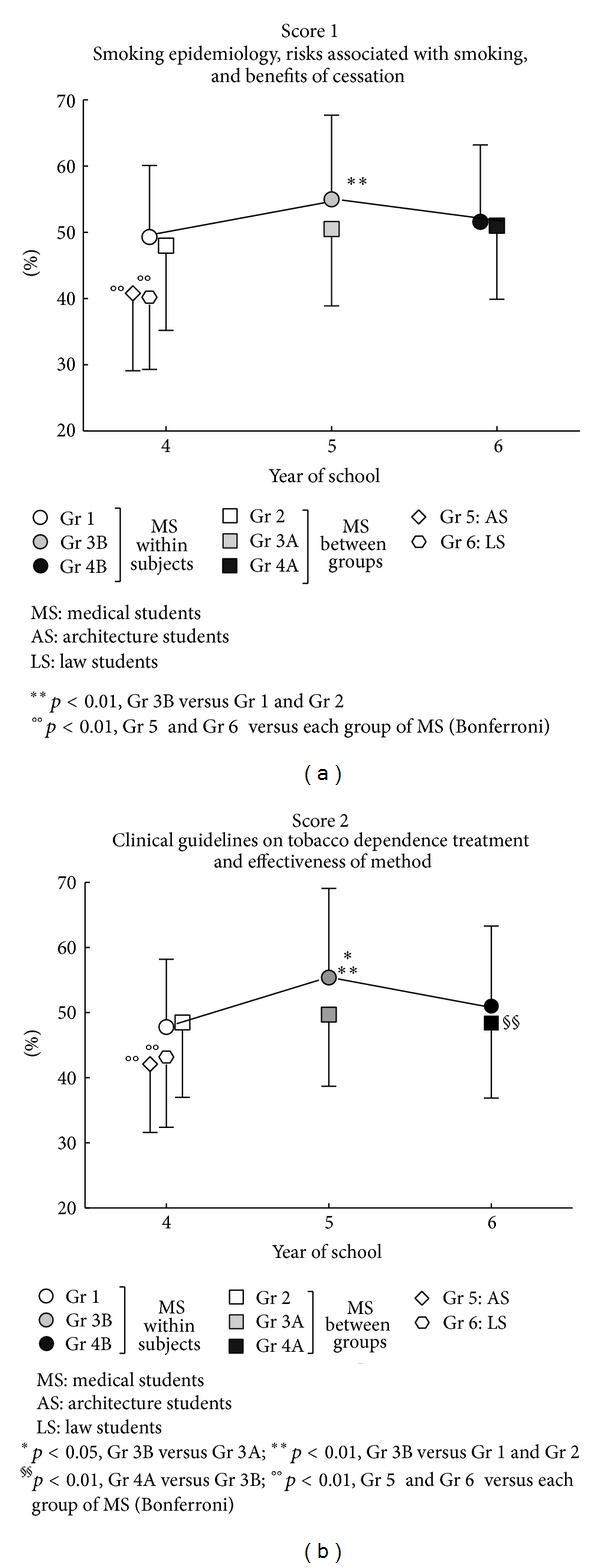
Scores of knowledge in medical students without or with a previous education intervention on nicotine dependence and in fourth year nonmedical students.

As shown in Figure 2, fourth year medical students had limited knowledge of the epidemiology of smoking, in terms of attributable morbidity and mortality, and of the benefits of stopping smoking (Score 1), before attending the educational intervention, with no statistically significant differences between groups. We also confirmed that knowledge of clinical guidelines on nicotine dependence treatment, perceived competence in both counseling and treating smokers was insufficient (Score 2), with no statistically significant differences between groups.

#### 3.3.2. Study Question 2

In order to address study question 2, only data obtained from fourth year students were included in the analysis. Medical students survey data were collected in 2010 (Group 1) and 2011 (Group 2) while nonmedical students data were collected in 2012 (Groups 5 and 6). Mean values for Score 1 were 48.9 ± 11.5% in medical students and 40.5 ± 11.4% in nonmedical students (*P* < 0.001; effect size *d* = 0.69). Respective values for Score 2 were 48.1 ± 10.8% and 42.6 ± 10.6% (*P* < 0.001; *d* = 0.50). These results suggest that the choice and attendance of a medical school are associated with marginal improvement in these parameters.

#### 3.3.3. Study Question 3

In 2011, there were statistically significant differences in knowledge levels between those who had attended the lecture in 2010 (Group 3B) and those who had missed it (Group 3A). This was true for Score 1 (55.0 ± 12.7% versus 50.5 ± 11.6%, *P* = 0.01; effect size *d* = 0.37) as well as for Score 2 (55.4 ± 13.7% versus 49.7 ± 11.0%, *P* = 0.001; effect size *d* = 0.46). However, no significant differences were observed in 2012 (i.e., two years after attending versus missing the lecture; see Figure 2 and Table 2).

## 4. Discussion

The present study confirms and extends our previous observation about the inadequate knowledge among medical students of nicotine dependence and provides two additional novel findings. Thus, our findings in the 2010 cohort that had previously been reported [20] were confirmed in a subsequent albeit smaller sample of fourth year medical students (Group 2). This is important since, due to a limited sample size in our earlier study, we were unable to exclude confounding of our results by selection bias. Moreover, we found that knowledge scores in nonmedical students were significantly lower than in medical students; however, the difference appeared relatively small when considering that the latter had already received three years of medical education. The knowledge levels observed in nonmedical students are likely to reflect general knowledge levels in well-educated young adults. A mean difference of only 10% points between medical and nonmedical students indicates a substantial failure of medical education in providing medical students with better knowledge of a fundamental issue of disease prevention than a general student population. Finally, medical students attending a lecture on nicotine dependence did better in a follow-up test one year later than students who had not been exposed to this intervention. Unfortunately, this difference between “intervention” and “control” groups was lost two years later.

The finding that a single lecture significantly improved the knowledge about tobacco-related issues one year later is of considerable interest and is consistent with similar results obtained in other medical disciplines. In particular, giving a single teaching lecture on a specific medical issue [24–26] has been found to permanently improve the ability to deal with those medical problems. Unfortunately, differences in knowledge seemed to be transient in as far as scores were back to baseline levels two years later. Yet, it is interesting to note that, even two years after the intervention, this group of students showed a smoking prevalence of 10.3% and the lowest FTND scores among groups. However, this result may also be explained by selection bias favouring students who were interested in the topic, thus being more motivated to complete the questionnaire again. Incidentally and in agreement with previous observations [27], all smoking students scored low at FTND; the lowest values were found in medical students.

Smoking prevalence among medical students was lower (16.9%) with respect to both the Italian population (20.7%) [3] and their colleagues of architecture and law school (26.3%). As smoking status was not biochemically validated, there is a possibility that smoking prevalence was underestimated in medical students. One potential explanation for this is selection bias in that smoking students might have been less likely to attend the lecture in the first place. Secondly, the students sampled may not be representative of all Italian medical students. Thirdly, according to the effect of social desirability, smoking medical students may have been more likely to misreport their smoking status as they felt it would be inappropriate for future physicians to be smoking. At the same time, our findings could actually reflect true smoking prevalence, as nonsmokers may be more likely to study medicine. Given the uncertainty associated with self-reports of smoking status, we refrained from conducting subgroup analyses or running statistical models including smoking status as a moderating variable.

As discussed above, our educational intervention that consisted of a single lecture on nicotine dependence was associated with higher knowledge levels one year after the intervention. A combination of educational and interactive training during medical school improves knowledge, attitude, and counselling skills on tobacco cessation and behavioural change [28, 29]. Role-playing and interaction with patients are equally effective and both represent more powerful learning tools than web-based learning with or without a teaching lecture [30]. Yet, overcrowded core curricula in many medical schools limit the possibility of extended training, and perhaps the most parsimonious strategy may consist in educating clinical teachers to mention tobacco toxicology whenever the possibility arises. This could be a cost-effective and efficient way of improving knowledge.

Thus, our results provide the rationale for studies comparing the effects of a single educational intervention with those yielded by a more comprehensive training in the health consequences of smoking. Interestingly, recent research has revealed that the choice of the educational method is far less important for student learning than summative assessments. As a consequence, medical students should undergo valid summative assessments of their knowledge of nicotine dependence [31].

In our opinion, the present study has five main limitations: (i) we included medical students from only four Italian universities; therefore, our sample is not fully representative of the entire population of Italian medical students; (ii) attrition substantially reduced to approximately one-fourth the number of students that were retested in the fifth and sixth years; thus, selection bias favoring the subsequent participation of students with higher interest levels in tobacco-related issues and higher motivation might have skewed our results; (iii) the sample size of architecture and law students was relatively small, questioning the representativeness of our findings in these groups; (iv) smoking status of participating students was only assessed by means of self-report so that smoking prevalence might have been underestimated; (v) we could not track individuals and their change in responses since we did not include identifying information.

## 5. Conclusions

In summary, this study revealed that Italian undergraduate medical students have marginally higher knowledge about smoking-related disease and methods to achieve cessation than students of nonmedical schools. Attending a lecture on nicotine dependence was associated with slightly better knowledge one year later, but the effect was moderate and short-lived. Greater efforts are needed to educate a generation of physicians that will have to deal with the consequences of the smoking epidemic in the 21st century.

## Figures and Tables

**Table 1:** Smoking habits, medical advice to quit, and intention to quit among 1191 university students.

	School							
	Medical						Architecture	Law
Year of observation	2010	2011	2011	2011	2012	2012	2012	2012
Group	1	2	3A	3B	4A	4B	5	6
Year of school	4	4	5	5 (Retest)	6	6 (Retest)	4	4
*n*	*439 *	*203 *	*113 *	*104 *	*207 *	*126 *	*122 *	*107 *
Smoking habits°								
Never smokers**	330 (75.2)	145 (71.4)	75 (68.2)	80 (76.9)	133 (64.3)	101 (80.2)	73 (59.8)	62 (57.9)
Current smokers**	67 (15.3)	38 (18.7)	21 (18.6)	14 (13.5)	49 (23.7)	13 (10.3)	32 (26.2)	28 (26.2)
Former smokers*	42 (9.6)	18 (8.9)	14 (12.7)	10 (9.6)	25 (12.1)	12 (9.5)	17 (13.9)	15 (14.0)
Mean age at onset of smoking among never smokers: years	16.5 ± 2.4	17.1 ± 2.7	17.2 ± 3.2	16.6 ± 2.5	17.4 ± 2.8	17.0 ± 3.5	16.8 ± 2.2	16.4 ± 1.7
(range)	(12–24)	(12–25)	(9–25)	(13–24)	(12–24)	(11–24)	(13–22)	(13–22)
Mean age of smoking cessation among former smokers: years	20.1 ± 2.4	20.9 ± 3.7	20.2 ± 4.2	21.3 ± 3.3	20.7 ± 3.5	20.3 ± 3.0	21.1 ± 2.7	20.5 ± 2.2
(range)	(15–24)	(15–31)	(14–30)	(15–25)	(14–30)	(15–24)	(17–29)	(18–27)
Characteristics of current smokers								
Fagerström score	1.3 ± 1.7	1.2 ± 1.6	1.5 ± 2.3	1.7 ± 1.9	1.9 ± 2.2	0.3 ± 0.7	2.0 ± 2.4	1.8 ± 1.9
(range)	(0–6)	(0–5)	(0–8)	(0–5)	(0–7)	(0–2)	(0–7)	(0–6)
Tried to quit and relapsed in the past	30 (44.8)	20 (54.1)	9 (42.9)	6 (42.8)	18 (36.7)	4 (30.8)	12 (37.5)	11 (39.2)
Tried to stop smoking during university	29 (43.3)	20 (54.1)	11 (52.4)	7 (50.0)	16 (32.7)	4 (30.8)	16 (50.0)	11 (39.3)
Tried in the last year to stop smoking and succeeded for 1 day or longer	36 (53.7)	25 (67.6)	15 (71.4)	10 (71.4)	27 (55.1)	9 (69.2)	20 (62.5)	16 (57.1)
Medical advice to quit (answered yes)								
“In the last year, did a doctor advise you to stop smoking?”	11 (16.4)	12 (32.4)	4 (19.0)	3 (21.4)	11 (22.4)	4 (30.8)	6 (18.8)	5 (17.9)
Intention to quit (answered yes)								
“Would you like to give up smoking altogether?”	40 (59.7)	24 (64.8)	11 (52.3)	9 (64.3)	25 (51.0)	5 (38.5)	18 (56.3)	21 (75.0)
Which of the following statements best describes your current intentions with regard to smoking?								
“I want to quit, but I'm not ready to try now.”	26 (38.8)	11 (29.7)	5 (23.8)	5 (35.7)	17 (34.7)	4 (30.8)	5 (15.6)	10 (35.7)
“I will continue to smoke for now.”	13 (19.4)	8 (21.6)	9 (42.8)	3 (21.4)	20 (40.8)	6 (46.2)	14 (43.4)	12 (42.9)

Data are expressed as *n* (%) or M (±SD); *P < .05 and **P < .01 Pearson's chi-squared test.

°Seven missing answers: two in Group 2, three in Group 3A, and two in Group 6.

**Table 2:** Mean scores for two sets of 14 grouped items in 1191 university students.

	School								*P* value^a^
	Medical						Architecture	Law
Year of observation	2010	2011	2011	2011	2012	2012	2012	2012	
Group	1	*2 *	3A	3B	4A	4B	5	6	
Year of school	4	4	5	5 (retest)	6	6 (retest)	4	4	
Total sample (n)	439	203	113	104	207	126	122	107	

Knowledge of smoking epidemiology, risks associated with smoking, and benefits of cessations: Score 1									
*n*	393	180	103	97	189	120	109	95	
Mean (SD)	49.3 (10.8)	47.9 (12.8)	50.5 (11.6)	55.0 (12.7)	51.0 (11.1)	51.6 (11.6)	40.8 (11.7)	40.2 (10.9)	<.001
Range (0–100)	17–87	17–87	23–70	27–87	23–80	20–83	17–77	20–67	

Knowledge of clinical guidelines on tobacco dependence treatment and effectiveness of method: Score 2									
*n*	391	182	103	98	191	118	105	92	
Mean (SD)	47.8 (10.4)	48.8 (11.5)	49.7 (11.0)	55.4 (13.7)	48.4 (11.5)	51.0 (12.3)	42.1 (10.5)	43.2 (10.8)	<.001
Range (0–100)	7–87	17–87	30–77	23–87	17–77	20–80	20–67	13–70	

*n*: number of subjects observed.

^a^Analysis of variance.
